# Sex Differences in Coronary Function Test Results in Patient With Angina and Nonobstructive Disease

**DOI:** 10.3389/fcvm.2021.750071

**Published:** 2021-10-14

**Authors:** Tijn P. J. Jansen, Suzette E. Elias-Smale, Stijn van den Oord, Helmut Gehlmann, Aukelien Dimitiriu-Leen, Angela H. E. M. Maas, Regina E. Konst, Niels van Royen, Peter Damman

**Affiliations:** Department of Cardiology, Radboudumc, Nijmegen, Netherlands

**Keywords:** ANOCA, coronary function test, microvascular dysfunction, vasospasm, sex, IMR, CFR

## Abstract

**Introduction:** Invasive coronary function testing (CFT) has become the recommended diagnostic tool to assess the various endotypes of coronary vasomotor dysfunction in patients with angina and no obstructive coronary artery disease (ANOCA), which has implications for therapy and prognosis. Although the expanding performance of CFT is leading to increased knowledge of coronary vasomotor dysfunction, little is known about sex-related differences in the results of comprehensive CFT.

**Methods:** We conducted a prospective study of all consecutive patients with ANOCA that underwent clinically indicated CFT in a tertiary interventional from February 2019 to February 2021. CFT consisted of acetylcholine testing to diagnose epicardial or microvascular spasm, and adenosine testing to diagnose CMD. CMD was defined as an index of microvascular resistance (IMR) ≥ 25 and/or coronary flow reserve (CFR) < 2.0.

**Results:** In total, 228 women and 38 men underwent CFT. No differences in traditional risk factors were seen, but women had a higher prevalence of migraine (45 vs. 14%, *p* = 0.001). Men more often had a history of percutaneous coronary intervention (12 vs. 49%, *p* = 0.001). We found no difference in clinical presentation. Coronary vasomotor dysfunction was present in 95% of men and 88% of women (*p* = 0.25), but males show more often epicardial spasm and less microvascular spasm than women (63 vs. 42% and 29 vs. 40% respectively, *p* = 0.039). Impaired CFR was more prevalent among females (6 vs 20%, *p* = 0.033). IMR [median of 23 (15–32) vs. 19 (13–25), *p* = 0.08] did not differ between the sexes.

**Conclusion:** Men undergoing CFT show a comparable prevalence of coronary vascular dysfunction as women. However, men have a higher prevalence of epicardial spasm and a lower prevalence of microvascular spasm compared with women. An impaired CFR was more often present in women, with an equally impairment of IMR.

## Introduction

Angina pectoris is one of the most common presenting symptoms in our healthcare system, and it is generally ascribed to obstructive coronary artery disease (CAD). However, in a large proportion of women (up to 70%) and a smaller proportion of men (about 30%) no obstruction of the coronary arteries is observed during coronary angiography (1). Despite, these patients have a worse prognosis then asymptomatic patients the absence of epicardial obstructive disease, cardiovascular outcome is unfavorable (1, 2). In the past few years it has been shown that the majority of these patients with so-called Angina and No Obstructive Coronary Artery Disease (ANOCA) have an alternative cause of ischemic heart disease, namely coronary vasomotor dysfunction (3). This condition is linked to increased cardiovascular risk, and impaired quality of life, and are a source of it leads to considerable health resource utilization (4–6). Although ANOCA is thought to be more prevalent in women and the majority of studies are performed in women (6–8), men are also affected by coronary vasomotor dysfunction (9, 10).

Coronary vasomotor dysfunction can be divided into different pathophysiological (endo) types namely, epicardial and/or microvascular vasospasm, and coronary microvascular dysfunction (CMD) (11). These endotypes can be distinguished by an invasive coronary function test (CFT), using acetylcholine (ACH) (or equivalent) to provoke coronary spasm and adenosine (ADE) to evaluate CMD (12). In healthy endothelium, ACH stimulates the endothelial cell production of vasodilator substances such as nitric oxide, which overrules the direct vasoconstrictor effects of ACH on the vascular smooth muscle cells (VSMCs). Although the exact mechanism is still unclear, it is thought that when endothelial dysfunction is present, this results in a nitric oxide depletion of the endothelial cells, leaving the VSMCs hyperreactive to vasoconstricting stimuli, resulting in coronary artery spasm (13). Adenosine testing enables the assessment of the coronary flow reserve (CFR), reflecting microvascular dilatory reserve capacity and the index of microvascular resistance (IMR), representing minimal microvascular resistance in hyperemic conditions. Both can be impaired in CMD.

Previous studies indicated that there might be differences in the prevalence of these endotypes between men and women (5, 14). However, no study to date has showed data on sex differences in patients who underwent comprehensive CFT, comprising both ACH and ADE testing to evaluate both epicardial and microvascular coronary function.

Elucidating sex differences in the prevalence of coronary vasomotor endotypes may lead to a better understanding of the pathophysiological mechanisms and treatment response and might enable improved patient-tailored treatment. In this paper we will evaluate sex differences in CFT characteristics including the prevalence of endotypes, differences in cardiovascular risk factors and symptoms.

## Methods

### Study Population

Prospective registry including all consecutive patients who were referred for CFT or ACH testing for suspected coronary vasomotor dysfunction by their treating cardiologist, at a large tertiary referral center (Radboudumc, Nijmegen, the Netherlands), between February 2019 to February 2021. In general, obstructive CAD was ruled out pre-CFT by invasive coronary angiography or Coronary Computed Tomography Angiography (CCTA). In line with current guideline recommendations, we did not exclude patients with previous PCI (12). The study protocol conforms to the international Conference on Harmonization/Good Clinical Practice standards and the Declaration of Helsinki. The protocol was approved by the Medical Ethics Review Committee of the Radboudumc, Nijmegen, the Netherlands. All patients gave written informed consent.

### Clinical Characteristics

Clinical data, including medical history, traditional and non-traditional cardiovascular risk factors (i.e., migraine, rheumatic disease) and symptom characteristics, were obtained from both the electronic patient file and an online patient questionnaire (13, 14). The online questionnaire included in-depth questions regarding angina characteristics, including the type of angina (e.g., chest pain or equivalent such as dyspnea), radiation and the provoking factor or initiating moment (at rest, during exercise, after exercise, during emotions/stress, at night). All data were stored in a secured database (CastorEDC).

### Coronary Function Test

The CFT was performed in accordance with the standardized protocol and as described earlier by Konst et al. (10) and Ong et al. (15). In short, patients were instructed to withhold all vasoactive medication and methylxanthine-containing substances, such as coffee and bananas, for 24–48 h before the procedure, depending on half-life time. First, a diagnostic CAG was performed to confirm the absence of obstructive CAD, defined as a visual stenosis of more than 50% in combination with an Fractional Flow Reserve (FFR) ≤ 0.80 (16). If no or non-obstructive coronary artery disease was present, CFT performed. Note that, when performed via the radial artery, no nitroglycerin was administered, only verapamil 2.5 mg.

#### Coronary Vasospasm Provocation With Acetylcholine (ACH)

Incremental doses of 2, 20, 100 and 200 μg of ACH were manually infused over a period of 1–3 min into the left coronary artery (LCA) through a guiding catheter. After each infusion, cine-images were obtained to assess the change in coronary diameter. Heart rate, blood pressure and a 12-lead ECG were continuously monitored. After the 200 μg dose or in case of clear epicardial spasm at a lower dose, 0.2mg nitroglycerin was injected into the LCA.

#### Coronary Microvascular Dysfunction With Adenosine (ADE)

Subsequently, using a guidewire with distal pressure and temperature sensors, the structural microvascular function was assessed using a bolus thermodilution method (17). First the resting mean transit time (Tmn) was determined by injections of 3–5 mL room temperature saline into the LAD artery, averaging at least three consecutive measurements. Next, adenosine (typically 140 μg/kg/min) was administered intravenously to induce steady state maximal hyperemia—and thereby minimal microvascular resistance—and at least three more injections of room temperature saline were recorded and averaged to determine the hyperemic Tmn. The coronary flow reserve (CFR) was determined by dividing the average resting Tmn by the average hyperemic Tmn (18). Microvascular resistance, measured as the index of microvascular resistance (IMR), was calculated as the distal pressure (Pd) at maximal hyperemia multiplied by hyperemic Tmn (19, 20). All measurements were automatically analyzed by dedicated software (Coroventis Coroflow, Uppsala, Sweden).

### Definitions

We defined coronary vasomotor dysfunction according to the underlying pathophysiological endotype into patients with or without coronary spasm (ACH+ vs. ACH–), and patients with or without CMD as measured with adenosine (ADE+ vs. ADE–) (19). CMD (ADE+) was present when measurements showed an abnormal CFR <2.0 and/or an abnormal IMR ≥ 25, as defined by current consensus documents (21). The definitions of epicardial or microvascular spasm (ACH+) were defined in line with these guidelines as follows (10, 21, 22): epicardial vasospasm was defined as a focal or diffuse epicardial coronary diameter reduction ≥90% in response to ACH, compared to the relaxed state after intracoronary nitroglycerin infusion, with a reproduction of (recognizable) symptoms and ischemic ECG changes. Microvascular spasm was diagnosed when the patient experienced the reproduction of recognizable symptoms with ischemic ECG changes, in the absence of ≥90% epicardial diameter reduction during ACH infusion (21). Ischemic ECG changes were defined as transient ST-segment elevation or depression of ≥0.1 mV, or ischemic T-wave changes, in at least two contiguous leads. Any inconclusive result in response to ACH (e.g., only reproduction of symptoms) was considered negative. A normal CFT was defined as both normal ACH and adenosine tests (ACH– and ADE–).

Furthermore, based on their combination of endotype of vasomotor dysfunction, the following definitions are used: isolated ACH+ (only epicardial or microvascular spasm), isolated ADE+ (only abnormal CFR/IMR), or both ACH+ and ADE+ (combined vasomotor dysfunction) (11).

### Statistical Analyses

Continuous data are presented as mean ± standard deviation (SD), or median and interquartile interval, where appropriate. We used the Kolmogorov-Smirnov test to check for normal distribution of data. Categorical data are presented as numbers (%). Differences between groups were assessed by an independent sample *t*-test for continuous data with a normal distribution. Otherwise, the nonparametric Mann-Whitney U test was used. Categorical data were compared with the use of Fisher's exact test. A two-sided *P* < 0.05 was considered statistically significant. All analyses were performed using SPSS Statistics version 25 (SPSS Inc., Chicago, IL, USA).

## Results

### Clinical Characteristics

We studied 266 patients, of whom 228 were females and 38 were males. Mean age was 58 ± 8, with no difference between the sexes, as shown in Table 1.

**Table 1:** Patient characteristics of patients undergoing coronary function testing according to sex.

	**Total** ***N* = 264**		**Female** ***N* = 228**		**Male** ***N* = 38**		***P*-value**
Age (years)	58	± 8	58	± 8	60	± 8	0.11
**Relevant medical history**							
History of MI	47	(18%)	36	(16%)	11	(30%)	**0.041**
History of PCI	45	(17%)	27	(12%)	18	(49%)	**0.001**
History of CVA/TIA/PAD	18	(7%)	18	(8%)	2	(5%)	0.83
Non-invasive ischemia detection test performed^a^	177	(71%)	147	(69%)	30	(83%)	0.09
Positive test result for ischemia	48	(27%)	39	(27%)	9	(32%)	0.58
**Cardiovascular risk factors**							
≥ 3 cardiovascular risk factors	136	(53%)	116	(53%)	20	(54%)	0.88
Adipose (BMI ≥ 25)	153	(58%)	127	(57%)	26	(68%)	0.18
Hypertension	116	(44%)	103	(45%)	13	(34%)	0.21
Dyslipidemia	91	(35%)	80	(35%)	11	(29%)	0.46
Diabetes	27	(10%)	20	(9%)	7	(17%)	0.07
Current/former smoker	154	(55%)	118	(52%)	26	(69%)	0.13
Premature CAD in first-degree relative	132	(50%)	116	(51%)	16	(43%)	0.36
**Other risk variables**							
Migraine	98	(40%)	93	(45%)	5	(14%)	**0.001**
Rheumatic disorder	44	(18%)	38	(18%)	6	(17%)	0.96
**Medication use**							
Betablocker	94	(35%)	81	(36%)	13	(34%)	0.88
Long acting nitrates	62	(23%)	46	(20%)	16	(42%)	**0.003**
CCS-antagonists	174	(65%)	155	(68%)	19	(50%)	**0.031**
Nicorandil	51	(19%)	42	(18%)	9	(24%)	0.45
ACE-i	44	(17%)	29	(13%)	15	(40%)	**0.001**
Aspirin	109	(41%)	84	(37%)	25	(66%)	**0.001**
Statin	136	(51%)	109	(48%)	27	(71%)	**0.008**
**Laboratory results**							
NT-proBNP (in pg/ml)	88	(41–138)	90	(40–140)	69	(9–130)	**0.014**
LDL cholesterol (in mmol/l)	2.38	(1.81–2.95)	2.46	(1.93–2.99)	1.91	(1.16–2.65)	**0.002**
CRP	3.7	± 18.5	4.09	± 19.9	1.40	± 1.8	0.62
CK	71	(49–93)	70	(52–88)	114	(70–148)	**0.001**
eGFR	84.8	± 9	84.3	± 9.4	88	± 5.1	**0.048**
**Angina characteristics**	*N* = 216		*N* = 189		*N* = 27		
Chest pain	179	(83%)	157	(83%)	22	(81%)	0.84
Dyspnea	145	(67%)	130	(69%)	15	(56%)	0.17
Symptoms nitrate-responsive (*n* = 158)	133	(84%)	117	(85%)	16	(76%)	0.28
Symptoms at rest	187	(87%)	166	(88%)	21	(78%)	0.25
Symptoms during exercise	163	(76%)	144	(76%)	19	(70%)	0.51
Symptoms exerted by emotion or stress	141	(65%)	131	(69%)	10	(37%)	**0.001**

*Values are mean ± SD, n (%), or median [interquartile interval]*.

a *In the absence of significant obstructive CAD*.

*MI, myocardial infarction; PCI, percutaneous coronary intervention; CVA, cerebrovascular accident; TIA, transient ischaemic attack; PAD, peripheral artery disease; BMI, body mass index; CAD, coronary artery disease; CCB, calcium channel blocker; ACE-I, angiotensin-converting enzyme inhibitor; CRP, C-reactive protein; LDL, low-density lipoprotein; CK, Creatinine Kinase; eGFR, estimated glomerular filtration rate. Significant values are in bold*.

Relevant medical history showed that males had a higher prevalence of a history of myocardial infarction and PCI (13 vs 30%, *p* = 0.041 and 12 vs. 49%, *p* = 0.001, respectively).

We observed no differences in cardiovascular risk factors between the groups, but females had a higher prevalence of migraine (45 vs. 14%, *p* = 0.001).

When evaluating pre-CFT medication use, we found several differences. Males were more often on long acting nitrates (20 vs. 42%, *p* = 0.003), ACE-inhibitors (13 vs 40%, *p* = 0.001), aspirin (37 vs. 66%, *p* = 0.001) and statins (48 vs. 71%, *p* = 0.008). Females tended to use more calcium channel antagonists (68 vs. 50%, *p* = 0.031) and both sexes were prescribed betablockers and nicorandil equally.

Anginal characteristic questionnaires revealed that both chest pain with radiation to arms, back or throat, and/or dyspnea were prevalent in all patients (83 and 67% respectively without differences between sexes), with good reported responsiveness to sublingual nitroglycerine (84%). With regard to the precipitating factor, both sexes experienced symptoms in rest or during exercise to equal extent. In females, however, symptoms were more often provoked by emotion or stress (69 vs. 37%, *p* = 0.001).

### Procedural Characteristics

Most CAGs were performed using a radial access site; in 17 (6%) patients a conversion to femoral access was necessary because of radial spasm (Table 2).

**Table 2:** Procedural characteristics according to sex.

	**Total** ***N* = 264**		**Female** ***N* = 228**		**Male** ***N* = 38**		***P*-value**
**Acetylcholine testing**							**0.039**
Epicardial spasm	118	(45%)	94	(42%)	24	(63%)	
Microvascular spasm	102	(39%)	91	(40%)	11	(29%)	
Negative	44	(17%)	41	(18%)	3	(8%)	
**Adenosine-induced hyperemia**							
Mean Rest Transit Time (sec)	0.91	(0.56–1.26)	0.86	(0.51–1.21)	1.22	(0.87–1.57)	**0.003**
Mean Hyperaemic Transit Time (sec)	0.26	(0.17–0.36)	0.25	(0.16–0.35)	0.27	(0.18–0.36)	0.17
CFR	3.3	(2.2–4.4)	3.3	(2.2–4.4)	3.95	(2.85–4.05)	**0.021**
CFR <2.0	46	(18%)	44	(20%)	2	6(%)	**0.033**
IMR	19	(13–25)	19	(13–25)	23	(15–32)	0.15
IMR ≥ 25	80	(32%)	64	(30%)	16	(44%)	0.08
FFR_ade_	0.90	(0.865–0.935)	0.90	(0.87–0.93)	0.87	(0.835–0.905)	**0.003**
**Prevalence of CMD**							**0.027**
Both CFR and IMR normal	145	(58%)	126	(58%)	19	(53%)	
Only abnormal CFR	27	(11%)	26	(12%)	1	(3%)	
Only abnormal IMR	61	(24%)	46	(21%)	15	(42%)	
Both CFR and IMR abnormal	19	(8%)	18	(8%)	1	(3%)	
**Combined results**							
Vasomotor dysfunction	237	(89%)	201	(88%)	36	(95%)	0.25
CMD (ADE+)	107	(43%)	90	(42%)	17	(47%)	0.53
Spasm (ACH+)	220	(83%)	185	(81%)	35	(92%)	0.12
CMD + spasm	89	(36%)	73	(34%)	16	(44%)	0.23
**Procedural results**							
**Final access site**							0.50
Radial	218	(83%)	188	(82%)	30	(79%)	
Femoral	45	(17%)	40	(18%)	8	(21%)	
Conversion	17	(6%)	15	(7%)	2	(5%)	0.69
**Complications**							
Temporary AV block due to ACH	20	(8%)	15	(7%)	5	(13%)	0.16
Serious adverse event	6	(2%)	6	(3%)	0	(0%)	0.29

*Values are n (%) or median [interquartile interval]*.

*FFR, fractional flow reserve; CMD, coronary microvascular dysfunction; AV, atrioventricular. Other abbreviations are similar to those used in Table 1. Significant values are in bold*.

Overall, there were no fatal, but five serious adverse events related to the procedure. These all happened in women. Two patients were affected by a coronary dissection, for which one PCI was performed and one was ameliorated with a plain balloon angioplasty. Three patients experienced a complication related to the access site (femoral artery stenosis after angioseal placement, BARC1 and BARC3 access site bleeding). In addition to that, during ACH infusions, 20 patients (7%) showed temporary AV-conduction disorders, this happened equally in both men and women.

### Coronary Vasospasm

**Figure 1:**
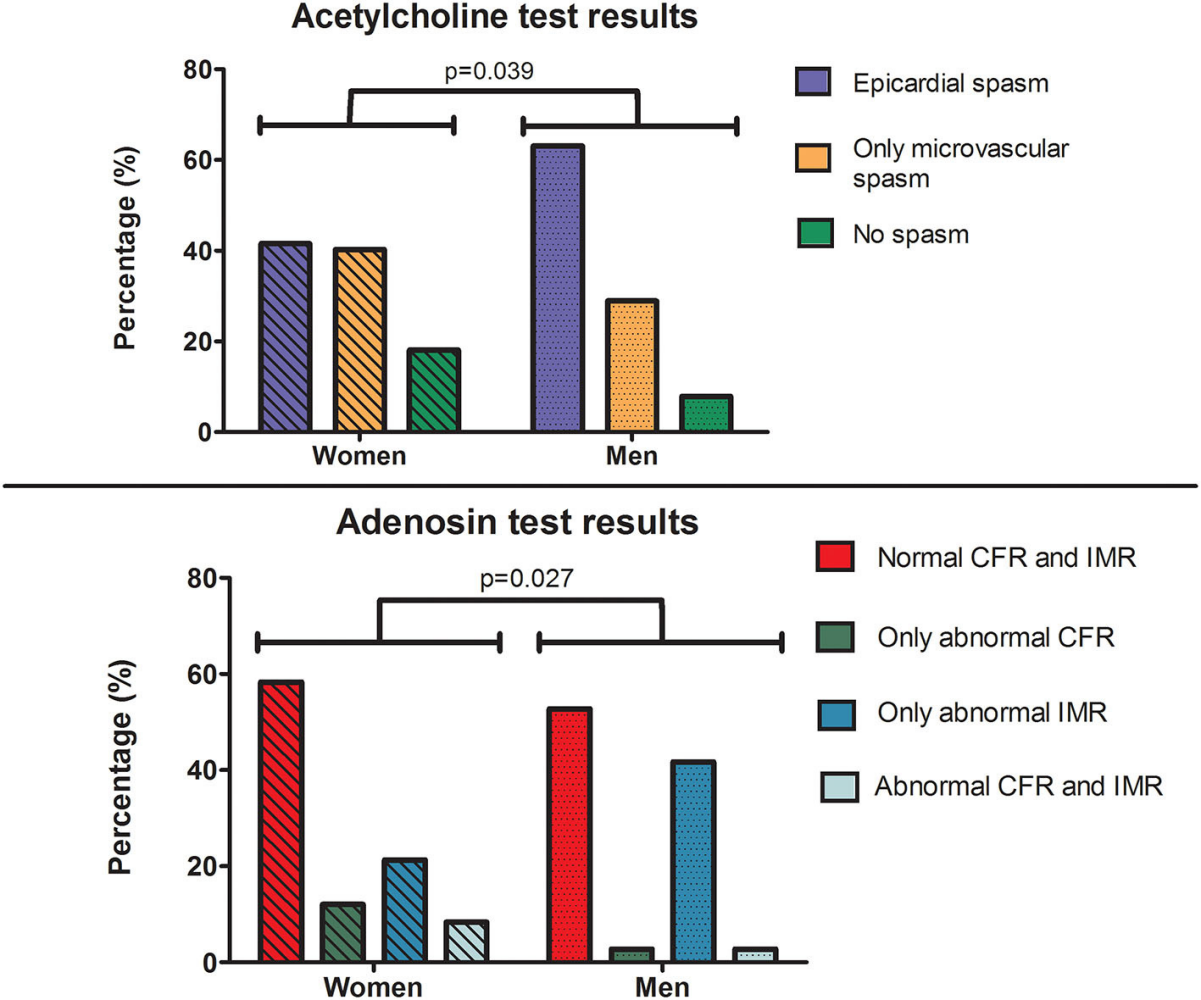
Central illustration: Acetylcholine and adenosine testing result differences between both genders. Top–Acetylcholine spasm testing in patients with angina and no obstructive coronary artery disease showed that men have a higher prevalence of epicardial spasm and a lower prevalence of microvascular spasm compared to women. Please note that the “epicardial spasm” group includes all patients who showed epicardial spasm at one of the dosages ACH, some of these patients showed signs of microvascular spams at a lower dosage of ACH. Bottom–Adenosine testing of microvascular function in patients with angina and no obstructive coronary artery disease showed that women have increased prevalence of impaired CFR and similar prevalence of impaired IMR. ACH, acetylcholine; ADE, adenosine; CFR, coronary flow reserve; IMR, index of microvascular resistance.

Of the 266 patients that completed ACH testing, 220 patients (83%) had coronary spasm (ACH+), 44 patients (16%) had no spasm and two tests were inconclusive (1%), as displayed in Table 2. Of the 220 patients with coronary spasm, 118 had epicardial spasm and 102 had microvascular spasm. We found a significant difference in the prevalence of both endotypes between both sexes. Males more often are affected with epicardial spasm and females are more often affected with microvascular spasm (epicardial 42 vs. 63% and microvascular 40 vs. 29%, *p* = 0.039) (Figure 1). Of the 120 patients with epicardial spasm, 77 patients showed diffuse spasm and 41 patients showed focal spasm, with no difference between sexes as displayed in Table 3. The average dose at which epicardial spasm was triggered was 126 μg and did not differ between the sexes. We note that in our population patients with prior PCI did not have a higher prevalence of epicardial spasm compared with patients without prior PCI (*p* = 0.134). In addition, when evaluating only the patients with no history of prior PCI the difference in prevalence between the sexes remains, as displayed in Table 4.

**Table 3:** Epicardial spasm characteristics according to sex.

**Epicardial spasm**	**Total** ***N* = 118**		**Female** ***N* = 94**		**Male** ***N* = 24**		***P*-value**
							0.52
Diffuse spasm	77	(65%)	60	(64%)	17	(71%)	
Focal spasm	41	(345%)	34	(36%)	7	(29%)	
**Triggering dosage (ACH)**							0.45
2 μg	3	(3%)	3	(3%)	0	(0%)	
20 μg	15	(13%)	10	(11%)	5	(22%)	
100 μg	53	(46%)	43	(46%)	10	(44%)	
200 μg	45	(39%)	37	(39%)	8	(35%)	
Mean maximum used dosage (μg)	126	± 66	128	± 65	117	± 69	0.49

*Values are n (%) or median [interquartile interval]*.

*ACH, acetylcholine*.

**Table 4:** Prevalence of spasm in patients with no history of prior PCI.

**No prior PCI**	**Female** ***N* = 199**		**Male** ***N* = 20**		***P*-value**
					**0.044**
Negative test	39	(20%)	1	(5%)	
Epicardial spasm	79	(40%)	13	(68%)	
Microvascular spasm	81	(40%)	5	(26%)	

*Values are n (%) or median [interquartile interval]. Significant values are in bold*.

### Coronary Microvascular Dysfunction

In total, 252 patients completed the adenosine microvascular function testing. The main reason for not performing adenosine measurements was a contra-indication to adenosine. Of the total group, median CFR was 3.3 (IQR: 2.2–4.4) and median IMR was 19 (IQR: 13–25). CMD was present in 107 patients (43%), in which an abnormal IMR was most prevalent (32% of all patients) (Central Illustration).

Males had a significantly higher CFR (median 3.3 vs 3.95, *p* = 0.021) and only two males had an abnormal CFR, while 44 females had an abnormal CFR (20 vs. 6%, respectively, *p* = 0.033). This was mainly due to a faster mean resting flow, as displayed by significantly shorter mean transit times at rest in females (0.86 vs. 1.22 s, *p* = 0.003), while the hyperemic flow did not differ between men and women (0.25 vs. 0.27 s, *p* = 0.20). There were no significant difference in the IMR results and the total prevalence of an abnormal IMR between both sexes, however men tend to have a higher prevalence of only impaired IMR (21 vs 42%).

### Combined Vasospasm and CMD

A third of all patients (*n* = 89/269) were affected by both vasospasm and CMD. This was equally divided between both sexes (34 vs. 44%, *p* = 0.23).

## Discussion

This is the first study evaluating sex differences in complete and comprehensive vasomotor function testing, comprising both vasospasm provocation testing and assessment of coronary microvascular dysfunction. The main findings are threefold. First, coronary vasomotor dysfunction is a common cause of angina in both men and women. Second, there are differences in the prevalence of endotypes between the sexes, with men more frequently affected by epicardial spasm and less frequently by microvascular spasm, when women have similar rates of both types of spasm. Third, with regards to coronary microvascular dysfunction, women have a higher mean resting flow (Tmn rest), a lower median CFR and are more frequently affected by an impaired CFR than men, while no difference in IMR was observed between the sexes. Finally, with regards to procedural characteristics, more complications were observed in female patients.

### Differences in Endotypes: Coronary Artery Spasm

In the present study, we demonstrated a higher prevalence of epicardial vasospasm in men vs. women, which is in line with currently available literature. Several studies reported a higher prevalence of epicardial vasospasm among men compared with women in selected populations undergoing ACH provocation testing (18, 20, 21). A possible explanation could be a higher extend of underlying (nonobstructive) atherosclerosis (18, 20, 22). Despite discordant reports in the last decades, earlier studies using intracoronary imaging suggest that epicardial vasospasm may be associated with mild atherosclerosis (23, 24). This is corroborated by the results in our cohort, with a lower FFR in male patients possibly indicating a higher atherosclerotic burden, and a more frequent history of previous PCI (Table 2). Atherosclerosis, an inflammatory process, leading to an imbalance of several vasodilator- and constrictor substances, provokes endothelial dysfunction (25). As mentioned in the introduction, this imbalance could lead to hyperreactive VSMC's and thus coronary vasospasm (13).

A second possible explanation might be the higher frequency of stent placement during PCI. In our study, 49% of males vs. 12% of females underwent a prior PCI. A stent may cause spasm due to direct toxic effect from the entrapped drug, delayed re-reendothelialization with inadequate endothelial coverage or acute or delayed hypersensitivity reaction to the polymer of the stent or the drug (26). Furthermore, mechanical provocation is possible, with the stent implantation causing interruption of vascular communication between the proximal and distal parts of the vessel because of stenting-induced dissection of the media and adventitia. This mechanical injury may predispose to vasoconstriction distal of the stent due to impaired vascular signaling (24). We note that in our population patients with prior PCI did not have a higher prevalence of epicardial spasm compared with patients without prior PCI (*p* = 0.134) and, when solely evaluation sex differences in patients without prior PCI, the differences remain (*p* = 0.044) despite the small amount of men. Larger samples are needed to elucidate the differences in coronary spasm between patients with and without previous PCI.

When evaluating the difference in sensitivity to ACH between the sexes, two recent studies demonstrated a higher susceptibility to acetylcholine in men. Sueda and Sakaue (27) found that in a Japanese population the mean maximum used ACH dose for provoking epicardial spasm in males was significantly lower than that in females. In addition to that, Pargaonkar et al. (28) found a greater response to intracoronary ACH at all doses for men, with a dose-response relationship with doses up to 200 μg, while women did not react significantly on ACH doses above 50 μg. In our study both females and males responded equally with dosages up to 200 μg acetylcholine.

### Difference in Endotypes: Coronary Microvascular Dysfunction

Our results show that women have a lower CFR than men, reflecting decreased microvascular dilatory reserve capacity, while no difference in IMR were observed, representing similar minimal microvascular resistance in hyperemic conditions. This finding is in line with the recent study by Kobayashi et al. (29), which showed that women have a lower median CFR in general. The lower CFR could be explained by a decreased augmentation of coronary flow from rest to hyperemia due to impaired microvascular dilatation, or due to a higher coronary resting flow in women. In our cohort, the lower CFR was mainly explained by higher baseline/resting flow.

The exact pathophysiological mechanism for the difference in microvascular function between men and women remains unclear. Similar as in FFR, which is also dependent on flow, there are well-known sex differences in several parameters that underlie CFR and IMR calculations, such as body size, artery size (and thus coronary blood flow), and myocardial mass (30). Additionally, the microcirculatory response to adenosine could differ by sex in patients with nonobstructive CAD. Finally, Crea et al. suggested a higher grade of diastolic dysfunction in women as a possible explanation (31). However, vice versa the higher grade of diastolic dysfunction could also be explained by the higher prevalence of CMD.

Importantly, from a prognostic perspective, a low CFR in women has been associated with worse outcome. Taqueti et al. (32) showed that, in a large population of patients referred for CAG for the evaluation of CAD, women had excess risk of cardiovascular events based on a higher prevalence of an impaired CFR compared to men. This was despite a lower pre-test clinical risk score, lower rates of prior myocardial infarction and/or PCI, and lower burden of obstructive CAD by invasive angiography.

In light of the connection between pathophysiological mechanism, prognosis and anginal symptoms much remains to be elucidated. Both a reduced CFR as well as an increased microvascular resistance have not been clearly linked to (microvascular) angina. As mentioned earlier, Kobayashi et al. proposed that an impaired CFR is mainly due to a relatively high resting flow. The influence of high resting flow, with diverse underlining pathophysiology, on prognosis and symptoms is not well clarified. Recently, Suppogu et al. (33) shed a new light on this association between high coronary rest blood flow and anginal symptoms. Within 259 women with suspected CMD baseline average peak velocity flow (bAPV) and hyperemic coronary blood flow after intracoronary adenosine was assessed with a doppler wire and CFR was calculated. It appeared that women with a higher than average bAPV had a significantly lower CFR, but more importantly had significantly more frequent angina and worse Seattle angina Questionnaire (SAQ-7) scores compared to women with a bAVP below the average. The authors hypothesize that an altered coronary autoregulation contributes to a higher bAPV, which can contribute to an impaired CFR. This could then lead to limit achieving adequate blood flow leading to impaired oxygen delivery and thereby more angina. The findings of our study corroborate to this hypothesis. We found that females have a higher mean resting flow and are more frequently affected by an impaired CFR. It would be interesting to see if higher mean resting flow could be linked to prognosis.

### Differences in Angina

We did not observe differences in angina characteristics between the sexes in this ANOCA population. All participants reported high rates of angina, both resting (87%) and during exercise (76%), regardless of their endotype of vasomotor dysfunction. Women did however reported stress or emotions as a triggering factor more often. Both stress (increased vagal tonus) and emotions (increased sympatic activity) have been shown to be able to induce spasm (13).

### Limitations

First, the amount of male patients that underwent CFT was low and represented around 15% of the cohort. This however resembles a real-life scenario and we were able to show significant differences, but subgroups were too small to confirm smaller differences. Second, this prospective study was performed with a cohort of ANOCA patients from single expertise center with a selected population of patients who were referred to CFT by their treating physician. This might have influenced the high yield of abnormalities we observed. On the other hand, studies including a less selected patient population also report a high prevalence of coronary vasomotor dysfunction in patients with ANOCA (3).

### Clinical Implications

Although we included patients with a history of previous PCI, which was more prevalent in men, and the study was dominated by women, the results of this study represent a non-selected population that from the daily practice. This reflects consecutive patients undergoing CFT in our tertiary referral center. The CFT is used to diagnose vasomotor dysfunction and to determine the endotype. The CORMICA trial has shown that the endotype identification has consequences for the subsequent choice of pharmacological therapy. Even though the yield of CFT was not different between our patients, the current study shows that there are differences in the observed endotypes, which has consequences for pharmacotherapy.

## Conclusion

Men undergoing CFT show similar a prevalence of coronary vasomotor dysfunction compared to women. Males, however, have a higher prevalence of epicardial spasm and a lower prevalence of microvascular spasm compared with women. Females more often show coronary microvascular dysfunction, with a higher prevalence of an impaired CFR, but similar prevalence of an impaired IMR.

## Data Availability Statement

The raw data supporting the conclusions of this article will be made available by the authors, without undue reservation.

## Ethics Statement

The studies involving human participants were reviewed and approved by CMO Arnhem-Nijmegen. The patients/participants provided their written informed consent to participate in this study.

## Author Contributions

TJ, SE-S, RK, AM, NR, and PD: forming and writing the manuscript. AD-L, SO, HG, NR, and PD: performing function test and collecting data. TJ, SE-S, and RK: collecting data. All authors contributed to the article and approved the submitted version.

## Conflict of Interest

The authors declare that the research was conducted in the absence of any commercial or financial relationships that could be construed as a potential conflict of interest.

## Publisher's Note

All claims expressed in this article are solely those of the authors and do not necessarily represent those of their affiliated organizations, or those of the publisher, the editors and the reviewers. Any product that may be evaluated in this article, or claim that may be made by its manufacturer, is not guaranteed or endorsed by the publisher.
